# A publicly available repository of ROH islands reveals signatures of selection in different livestock and pet species

**DOI:** 10.1186/s12711-020-00599-7

**Published:** 2021-01-04

**Authors:** Wim Gorssen, Roel Meyermans, Steven Janssens, Nadine Buys

**Affiliations:** Livestock Genetics, Department of Biosystems, KU Leuven, Kasteelpark Arenberg 30, Box 2472, 3001 Leuven, Belgium

## Abstract

**Background:**

Runs of homozygosity (ROH) have become the state-of-the-art method for analysis of inbreeding in animal populations. Moreover, ROH are suited to detect signatures of selection via ROH islands and are used in other applications, such as genomic prediction and genome-wide association studies (GWAS). Currently, a vast amount of single nucleotide polymorphism (SNP) data is available online, but most of these data have never been used for ROH analysis. Therefore, we performed a ROH analysis on large medium-density SNP datasets in eight animal species (cat, cattle, dog, goat, horse, pig, sheep and water buffalo; 442 different populations) and make these results publicly available.

**Results:**

The results include an overview of ROH islands per population and a comparison of the incidence of these ROH islands among populations from the same species, which can assist researchers when studying other (livestock) populations or when looking for similar signatures of selection. We were able to confirm many known ROH islands, for example signatures of selection for the *myostatin* (*MSTN*) gene in sheep and horses. However, our results also included multiple other ROH islands, which are common to many populations and not identified to date (e.g. on chromosomes D4 and E2 in cats and on chromosome 6 in sheep).

**Conclusions:**

We are confident that our repository of ROH islands is a valuable reference for future studies. The discovered ROH island regions represent a unique starting point for new studies or can be used as a reference for future studies. Furthermore, we encourage authors to add their population-specific ROH findings to our repository.

## Background

Runs of homozygosity (ROH) are long continuous homozygous stretches in the genome and are formed by the combination of two identical haplotypes in an individual [1]. Broman and Weber [2] first identified these long homozygous segments in the human genome and Gibson et al. [3] described their potential for inbreeding assessment. A genomic inbreeding coefficient based on ROH (F_ROH_) was first defined by McQuillan et al. [4]. Since 2010, analysis of ROH has become a standard approach to study inbreeding and detect signatures of selection in animal populations with the first reported studies in 2010 for cattle [5], in 2010 for dogs [6], in 2012 for pigs [7], in 2013 for horses [8], in 2014 for goats [9], in 2015 for sheep [10], in 2016 for cats [11] and in 2020 for water buffaloes [12]. Moreover, ROH analyses are complementary to genome-wide association studies (GWAS), inbreeding depression studies, genomic prediction and detection of deleterious variants and population-specific major genes [1, 13].

ROH analyses allow the accurate estimation of F_ROH_ on both the population and the individual level. It is commonly accepted that short ROH are indicators of distant consanguinity, whereas long ROH are more likely the result of recent inbreeding [14]. However, ROH may also result from small inversions that suppress recombination [1] or demographic events [15], such as a population bottleneck, genetic drift, or (artificial) selection. Moreover, it has been shown that ROH are more likely to arise in genomic regions with a low recombination rate and high linkage disequilibrium (LD), such as for the X-chromosome and near the centromere of chromosomes [1, 16]. In addition, copy number variants (CNV) and/or coverage gaps may lead to artefacts in ROH analyses [17]. The effect of gaps in SNP coverage can be reduced by adjusting the parameters set for the ROH analysis, as discussed by Meyermans et al. [18].

ROH facilitate the investigation of highly inbred genomic regions within a population, first referred to as ROH islands by Nothnagel et al. [19]. These ROH islands can provide important insights into the studied population and are likely to be signatures of positive selection due to LD [15, 19–21]. However, currently many populations have not been studied for the identification of ROH islands, although several large online genotype datasets are available for various species. Furthermore, even when such populations have been investigated for ROH islands, it is often difficult to compare the results between studies, for example because of differences in analysis methods or detection criteria.

In this paper, we provide an overview of ROH islands in 442 populations (18,633 individuals) from eight animal species (cat, cattle, dog, goat, horse, pig, sheep and water buffalo) using medium-density SNP data, which were all analyzed using a standardized protocol. The outcome and R-script of our analyses are made online available and can be used as a reference for future studies (https://doi.org/10.17605/OSF.IO/XJTKV). Since ROH islands are potential signatures of selection, overlapping ROH islands across populations and species are a valuable tool in comparative genomic studies and may reveal important genetic regions.

## Methods

Medium-density SNP data from eight species (cat, cattle, dog, goat, horse, pig, sheep, and water buffalo) and 797 populations were collected from online available datasets (Table 1). Detailed information and background on these data are described in the corresponding studies.

**Table 1:** Data overview per species

Animal species	Populations before QC	Animals before QC	Populations after QC	Animals after QC	SNPs before QC	References
Cat	47	2078	26	1657	58,888	[23]
Cattle	144	4103	50	2263	45,926	[28, 49–58]
Dog	146	5406	49	4414	160,432	[24]
Goat	143	4653	96	4327	49,943	[25, 47]
Horse	37	795	35	774	50,042	[26, 48]
Pig	146	2113	78	1438	52,783	[27]
Sheep	118	3609	100	3490	52,413	[28–33]
Water buffalo	16	346	8	270	53,830	[34]
Total	797	23,103	442	18,633		

The number of populations and individuals are shown for the raw datasets and after applying quality control. The number of autosomal SNPs available per species before quality control is also shown. The main cause for excluding a population was a lack of a sufficient number of individuals (n < 15)

*QC* quality control, *SNPs* single nucleotide polymorphisms

Quality control and ROH analyses were performed using the PLINK v1.9 software [22]. Each population was subjected to the following quality control (PLINK commands in brackets). For SNPs, only autosomal SNPs were retained, and neither minor allele frequency pruning (--maf), no Hardy–Weinberg equilibrium test (--hwe), nor LD pruning were performed [18]. Individual call rate was set to 0.90 (--mind 0.10) and possibly duplicated individuals were removed (--genome; PI_HAT > 0.95). Minimal SNP call rate was set to 0.95 (--geno 0.05) and only populations with more than 15 individuals were retained after quality control.

For the ROH analysis (--homozyg), no heterozygous SNP was allowed (--homozyg-window-het and --homozyg-het) and one SNP could be missing (--homozyg-window-missing) [35, 36]. The minimal number of SNPs per window (--homozyg-window-snp) and in the final ROH segment (--homozyg-snp) were breed specifically calculated by the L-parameter [18, 35, 37] and a window had a minimal size of 1000 kb (--homozyg-kb). The density was set to 150 kb/SNP, thus 1 SNP every 150 kb (--homozyg-density), the maximal gap was 1000 kb (--homozyg-gap), and the window threshold set to two outer SNPs [18]. Average SNP density was at least one SNP per 55 kb for all populations. Consequently, genome coverage was higher than 97% for all breeds, which means that the given settings allowed ROH detection for more than 97% of the autosomal genome.

ROH incidence was calculated as the percentage of animals with a SNP within an ROH segment for a given population and were visualised via Manhattan plots using the qqman package [38]. ROH islands were defined as regions where SNPs had a P-value for ROH incidence higher than a population specific threshold. This threshold was calculated based on standard normal z-scores derived from the distribution of ROH incidences. The top 0.1% of SNPs with a P-value higher than 0.999 using a z-score table for ROH incidence were considered to form ROH islands, as specified by Purfield et al. [15] and Gorssen et al. [39]. As an additional restriction, the minimal threshold for detection of ROH islands was set to 30%, which means that a ROH has to be present in at least 30% of the population to be included in a ROH island. For populations with high levels of inbreeding (e.g. Boxer dogs with a mean F_ROH_ of 45%), no SNP reached a P-value > 0.999 for ROH incidence. Therefore, a maximal threshold for ROH island detection was set at 80%, meaning that all ROH with an incidence higher than 80% were marked as ROH islands.

## Results

An R-script was developed for standardized breed-by-breed quality control and ROH analysis. This script uses a PLINK-format genotype file (.bim,.bed and.fam), with a unique family ID (FID) for each population. First, parameter settings are specified for quality control and ROH analysis. Second, the R-script performs quality control and a ROH analysis per population (FID). Third, the script creates Manhattan plots based on ROH incidence per SNP for every investigated population, and a summary table. For the 442 populations studied here, all figures and the R-script are deposited at Open Science Framework (OSF) (https://doi.org/10.17605/OSF.IO/XJTKV).

**Fig. 1:**
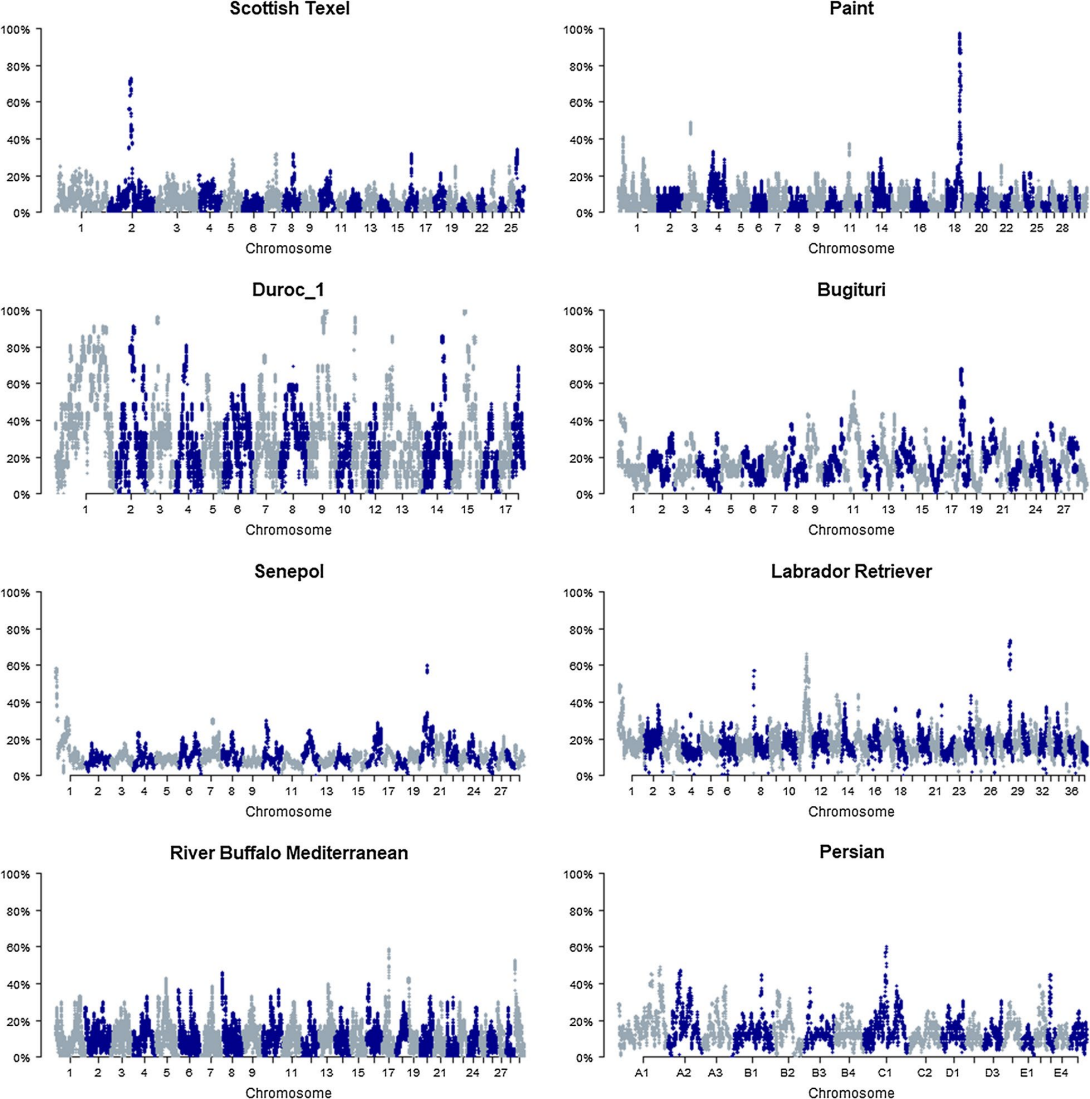
Incidence plots of SNPs in ROH for a population of each species studied in this analysis. Incidence plots of SNPs in ROH are given for a population of sheep (ScottishTexel), horse (Paint), pig (Duroc_1), goat (Bugituri), cattle (Senepol), dog (Labrador Retriever), water buffalo (Mediterranean River Buffalo) and cat (Persian). Clear ROH islands are visible: for example for Scottish Texel sheep (OAR2) and Paint horses (ECA18) around the *MSTN* gene. Details of ROH islands and populations are in Additional file 1: Tables S1, Additional file 2: Table S2, Additional file 3: Table S3, Additional file 4: Table S4, Additional file 5: Table S5, Additional file 6: Table S6, Additional file 7: Table S7, Additional file 8: Table S8, Additional file 9: Table S9, Additional file 10: Table S10, Additional file 11: Table S11, Additional file 13: Table S13, Additional file 14: Table S14, Additional file 15: Table S15, Additional file 16: Table S16

**Fig. 2:**
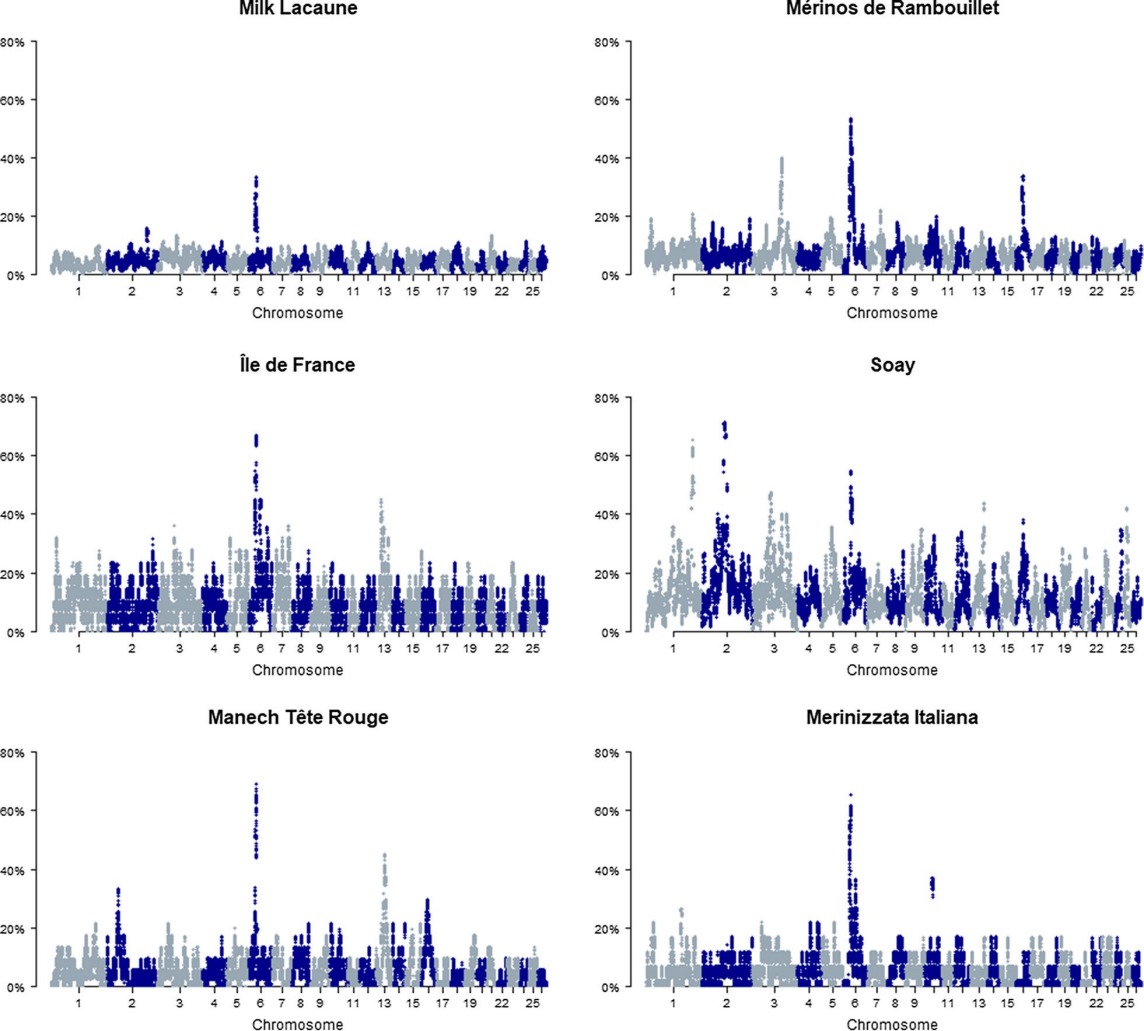
Incidence plots of SNPs in ROH for six sheep populations. These incidence plots show a remarkable ROH island on OAR6 around 38 Mb. This ROH island was seen in 15 of the studied sheep populations. Details of ROH islands and populations are shown in Additional file 13: Table S13, Additional file 14: Table S14

Figures 1 and 2 show an example of the ROH incidence plots. These Manhattan plots provide a quick overview of population-specific baseline ROH levels and ROH islands. For example, in Paint horses, ROH incidence levels are generally low (0 to 15%) but one remarkable ROH island is observed on *Equus caballus* chromosome (ECA)18 at 68 Mb. This ROH island was found in 23 out of the 24 studied horses in the Paint population (Fig. 1). Duroc pigs have higher baseline ROH levels (0 to 60%), with several ROH islands having an incidence higher than 80% (Fig. 1). To investigate ROH islands in more detail, we created tables with ROH island locations (bins of one Mb) per population, which are in Additional file 1: Tables S1, Additional file 2: Table S2, Additional file 3: Table S3, Additional file 4: Table S4, Additional file 5: Table S5, Additional file 6: Table S6, Additional file 7: Table S7, Additional file 8: Table S8, Additional file 9: Table S9, Additional file 10: Table S10, Additional file 11: Table S11, Additional file 12: Table S12, Additional file 13: Table S13, Additional file 14: Table S14, Additional file 15: Table S15, Additional file 16: Table S16 and in the repository. As an example, detailed information on the ROH islands per breed and chromosomal region for cats is provided in Table 2. These figures and tables can be used to detect overlapping ROH island regions in multiple populations. In cats, for example, 19 out of the 26 studied populations (73%) have a ROH island on chromosome B3 around 27–28 Mb (Table 2). In sheep, 15 of the 100 studied populations show a ROH island on *Ovis aries* chromosome (OAR)6 at ~ 38 Mb (Fig. 2, Additional file 13: Table S13 and Additional file 14: Table S14).

**Table 2:** ROH island regions (bins of one Mb) for 26 cat populations listed per chromosome

Population	Chromosome																	
	A1	A2	A3	B1	B2	B3	B4	C1	C2	D1	D2	D3	D4	E1	E2	E3	F1	F2
Abyssinian				34–42		27–30			65–91									
American Curl			17–18			143–146										15–41		
Bengal			87–90				82–89	39–40			60–77				37–38			
British Shorthair	83–87					24–30	32–37						33–33					
Burmese				35–49		22–30				26–55		28–32						
Colony						25–25									37–38			
Devon Rex							35–83											
Domestic						26–29												
LaPerm	83–84					27–29							31–34		25–38			
Lykoi						26–29									23–38			
Maine Coon	0–4					27–28							35–38			15–17		
Munchkin						25–29							30–33					
Norwegian Forest Cat	127–129	98–99				23–30	33–35								37–38			
Oriental	83–87					23–30	32–34	166–174	91–93									
Oriental Toygers	157–159		130–131			27–30	114–119					27–30			49–49			
Persian	160–203	58–61		149–150				104–112								15–17		
Peterbald	181–193					23–58		166–168	91–122									
Ragdoll	54–88			40–61		26–29				46–46								
Scottish Fold		101–103	49–50			25–30									37–39			
Selkirk Rex						24–29		103–110							37–40			
Siamese	152–186		49–52		73–73	25–30	32–34	166–174	91–93	43–45; 92–92								
Siberian	186–186					24–30							33–33					
Sphynx									136–140						53–57	15–17		
Tenessee Rex						25–31				22–41								
Turkish Van	34–34												31–33					
Wildcat																		

Within each cell of the table, the size of genomic region(s) with a ROH island is indicated in Mb. ROH islands were detected for multiple populations on e.g. chromosome B3 (25–29 Mb), E2 (37–38 Mb) and D4 (31–33 Mb)

## Discussion

We created a repository of ROH-islands for 442 populations from eight animal species (cat, cattle, dog, goat, horse, pig, sheep and water buffalo). These results are available online via OSF (https://doi.org/10.17605/OSF.IO/XJTKV) and examples are provided in Figs. 1 and 2, Table 2 and Additional file 1: Tables S1, Additional file 2: Table S2, Additional file 3: Table S3, Additional file 4: Table S4, Additional file 5: Table S5, Additional file 6: Table S6, Additional file 7: Table S7, Additional file 8: Table S8, Additional file 9: Table S9, Additional file 10: Table S10, Additional file 11: Table S11, Additional file 12: Table S12, Additional file 13: Table S13, Additional file 14: Table S14, Additional file 15: Table S15, and Additional file 16: Table S16.

For many populations, the figures of ROH incidence show interesting ROH islands. For example, previously known ROH islands are observed around the *myostatin* (*MSTN*) gene in Texel sheep (OAR2, 129 Mb) and Paint and Quarter horses (ECA18, 66 Mb) (Fig. 1) and Additional file 9: Table S9, Additional file 10: Table S10, Additional file 13: Table S13, and Additional file 14: Table S14. Both Purfield et al. [15] and Fariello et al. [40] found a signature of selection in the region spanning the *MSTN* gene in Texel sheep using different methods [15, 40]. Petersen et al. [26] showed the existence of a clear signature of selection in the genomic region around *MSTN* in Paint and Quarter Horses using F_ST_-based statistics [26]. *MSTN* is a major gene involved in muscle development. In sheep, selection on conformation has apparently led to a common ROH island, spanning the *MSTN* gene. In cats, we found that 19 of the 26 populations showed a ROH island on chromosome B3 between 20 and 30 Mb (Table 2). Montague et al. [41] found strong signatures of selection in cats in these regions on chromosome B3 using F_ST_-based analysis [41]. They suggested that the *ARID3B* gene—which is known to impact neural crest cell survival—might be the driving factor for the selection signature on chromosome B3 and they linked it to the domestication syndrome hypothesis [42].

Although the above-mentioned ROH islands had an assumed/known underlying biological factor driving positive selection, we detected multiple ROH islands in different species without a clear link to the underlying (biological) mechanisms, so far. These ROH islands might indicate genetic regions under positive selection, although they could also be the result of population bottlenecks, regions with repressed recombination, or artefacts caused by CNV or SNP gaps [1, 13, 17]. We observed that ROH and ROH islands occured more frequently near the centromere of chromosomes, possibly due to a difference in recombination levels [7, 17]. We minimized ROH artefacts that are caused by large SNP gaps, by optimizing ROH detection in PLINK as described in Meyermans et al. [18], in particular by adjusting the minimal SNP density for ROH detection. However, hemizygous deletions that are especially large might still resemble a ROH signal in SNP data [1, 4, 17]. To address this and also the possible interference between ROH and CNV via SNP data, raw genotyping data (e.g. Illumina final report files) are required. These are often not available for such large datasets and could not be examined in this study. Moreover, ascertainment bias might also impact ROH analysis as genotyping arrays can be less suitable for populations that are only distantly related to the populations used for array development. In this study, ascertainment bias was minimized by calculating the L-parameter, which takes potential heterozygosity differences into account [18, 35, 37] and by setting the minimum ROH length to 1000 kb. Furthermore, we would like to note that the presence of a ROH island does not implicate an identical underlying haplotype. For example, in the context of selection, ROH are likely to arise in genomic regions that contain important genes, but the underlying genotypes might differ among populations and individuals [39].

Regardless of their origin, ROH islands can provide a valuable clue for future research. For example, 15 sheep populations in this study show a ROH island on OAR6 at 38 Mb (Fig. 2). OAR6 appears to harbour multiple genes that are linked with milk production in sheep, but we also found several QTL for fat-tail, growth and bone-related traits near the region around 38 Mb [43, 44]. As another example, seven cat populations showed a ROH island on chromosome E2 (37–38 Mb) and six cat populations on chromosome D4 (33 Mb) (Table 2). However, to our knowledge, these regions have never been reported in the literature as regions of interest. Therefore, these overlapping ROH islands can be a starting point for researchers investigating signatures of selection but also for studies on islands of speciation, recombination hot- and coldspots and population history. Furthermore, researchers can compare the outcome of their (ROH) studies with our repository. Comparing (ROH) results among studies reported in the literature is often difficult, due to differences in quality control, software and parameter settings. The advantage of our repository is that it combines information on multiple populations and species which were analysed using a standardized method.

To define ROH islands, we implemented a population-specific threshold based on the ROH incidence distribution, which allows a comparison of ROH islands across different populations. In this study, we used PLINK for ROH detection as it is still the most frequently used software for these analyses [13]. Especially when using medium-density SNP data, it is essential to optimize (PLINK) ROH detection settings [18]. For example, the default value for density setting in PLINK is one SNP per 50 kb, which is higher than the mean density for our sheep (one SNP per 50.5 kb) and cattle (one SNP per 54.5 kb) SNP data even before quality control. Thus, using the default PLINK values can dramatically underestimate the number of ROH detected. Our recommendation for future studies using PLINK is to carefully consider these parameters, to make sure results are as correct and comparable as possible. To facilitate this, we share our R-script with other researchers upon proper citation (https://doi.org/10.17605/OSF.IO/XJTKV). We would like to draw attention to the fact that the dog data had a higher average SNP density (one SNP per 14 kb). However, differences in SNP density were accounted for by following Meyermans et al. [18]. Besides PLINK, other algorithms are also available for ROH detection, for example RzooRoH [45, 46]. Results obtained by using these software can also be included in our collection, since differences between rule-based (e.g., PLINK) and model-based (e.g., RzooRoH) approaches should be small when performed correctly on medium-density SNP data [39, 45, 46].

## Conclusions

We have shown that important ROH islands can be detected by scanning multiple populations simultaneously for ROH islands using a standardized detection method. We provide our script for standardized ROH island analyses and make all the results publicly available via OSF (https://doi.org/10.17605/OSF.IO/XJTKV). By sharing our results, our aim is to give researchers a useful reference to compare with their own analyses or to provide a unique starting point to investigate specific signatures of selection. Moreover, we encourage authors of future ROH studies to add their Manhattan plots of ROH incidence to our collection. We strongly believe that this ROH island repository will be very valuable for comparisons with future (ROH) studies or as a starting point for new studies.

## Supplementary Information

**Additional file 1: Table S1.** ROH island regions (bins of one Mb) for the studied cat populations: overview for all chromosomes. Within each cell, the genomic region(s) with an ROH island are given (in Mb).

**Additional file 2: Table S2.** ROH island regions (bins of one Mb) for the studied cat populations: details per chromosome. Within the Excel file, there is one tab for each chromosome with ROH island details per population. Cells are marked with an ‘x’ if an ROH island appeared in the specific region.

**Additional file 3: Table S3.** ROH island regions (bins of one Mb) for the studied cattle populations: overview for all chromosomes. Within each cell, the genomic region(s) with an ROH island are given (in Mb).

**Additional file 4: Table S4.** ROH island regions (bins of one Mb) for the studied cattle populations: details per chromosome. Within the Excel file, there is one tab for each chromosome with ROH island details per population. Cells are marked with an ‘x’ if an ROH island appeared in the specific region.

**Additional file 5: Table S5.** ROH island regions (bins of one Mb) for the studied dog populations: overview for all chromosomes. Within each cell, the genomic region(s) with an ROH island are given (in Mb).

**Additional file 6: Table S6.** ROH island regions (bins of one Mb) for the studied dog populations: details per chromosome. Within the Excel file, there is one tab for each chromosome with ROH island details per population. Cells are marked with an ‘x’ if an ROH island appeared in the specific region.

**Additional file 7: Table S7.** ROH island regions (bins of one Mb) for the studied goat populations: overview for all chromosomes. Within each cell, the genomic region(s) with an ROH island are given (in Mb).

**Additional file 8: Table S8.** ROH island regions (bins of one Mb) for the studied goat populations: details per chromosome. Within the Excel file, there is one tab for each chromosome with ROH island details per population. Cells are marked with an ‘x’ if an ROH island appeared in the specific region.

**Additional file 9: Table S9.** ROH island regions (bins of one Mb) for the studied horse populations: overview for all chromosomes. Within each cell, the genomic region(s) with an ROH island are given (in Mb).

**Additional file 10: Table S10.** ROH island regions (bins of one Mb) for the studied horse populations: details per chromosome. Within the Excel file, there is one tab for each chromosome with ROH island details per population. Cells are marked with an ‘x’ if an ROH island appeared in the specific region.

**Additional file 11: Table S11.** ROH island regions (bins of one Mb) for the studied pig populations: overview for all chromosomes. Within each cell, the genomic region(s) with an ROH island are given (in Mb).

**Additional file 12: Table S12.** ROH island regions (bins of one Mb) for the studied pig populations: details per chromosome. Within the Excel file, there is one tab for each chromosome with ROH island details per population. Cells are marked with an ‘x’ if an ROH island appeared in the specific region.

**Additional file 13: Table S13.** ROH island regions (bins of one Mb) for the studied sheep populations: overview for all chromosomes. Within each cell, the genomic region(s) with an ROH island are given (in Mb).

**Additional file 14: Table S14.** ROH island regions (bins of one Mb) for the studied sheep populations: details per chromosome. Within the Excel file, there is one tab for each chromosome with ROH island details per population. Cells are marked with an ‘x’ if an ROH island appeared in the specific region.

**Additional file 15: Table S15.** ROH island regions (bins of one Mb) for the studied water buffalo populations: overview for all chromosomes. Within each cell, the genomic region(s) with an ROH island are given (in Mb).

**Additional file 16: Table S16.** ROH island regions (bins of one Mb) for the studied water buffalo populations: details per chromosome. Within the Excel file, there is one tab for each chromosome with ROH island details per population. Cells are marked with an ‘x’ if an ROH island appeared in the specific region.

## Data Availability

The datasets analysed during the current study are available in the following repositories. *Cat* Gandolfi et al. [23]. Data available on: https://www.nature.com/articles/s41598-018-25438-0#Sec26. *Cattle* Sempéré et al. [28]. Data available on: http://widde.toulouse.inra.fr/widde/. Batch selection of all populations with SNP data from Illumina Bovine SNP50v1, Illumina Bovine SNP50v2 and Illumina BovineHD. Only common markers (46,387) were selected from all chromosomes. *Dog* Shannon et al. [24]. Data available on: https://datadryad.org/stash/dataset/doi:10.5061/dryad.v9t5h. *Goat* Colli et al. [25] and data from Bertolini et al. [47]. Dryad Digital Repository. https://doi.org/10.5061/dryad.v8g21pt. *Horse* Petersen et al. [26] and data from Petersen et al. [48]. NAGPR Community Data Repository. https://www.animalgenome.org/repository/pub/UMN2013.0125/. *Pig* Yang et al. [27]. Data at the Dryad Digital Repository. https://doi.org/10.5061/dryad.30tk6. *Sheep* Sempéré et al. [28]. Data available on: http://widde.toulouse.inra.fr/widde/. Batch selection of all populations with SNP data from Illumina OvineSNP50v1 and AgResearch OvineHD. Only common markers (42,439) were selected from all chromosomes. *Water buffalo* Colli et al. [34]. Data available at: https://datadryad.org/stash/dataset/doi:10.5061/dryad.h0cc7.
